# The Association of *Helicobacter pylori* Eradication with the Occurrences of Chronic Kidney Diseases in Patients with Peptic Ulcer Diseases

**DOI:** 10.1371/journal.pone.0164824

**Published:** 2016-10-20

**Authors:** Jiunn-Wei Wang, Chien-Ning Hsu, Wei-Chen Tai, Ming-Kun Ku, Tsung-Hsing Hung, Kuo-Lun Tseng, Lan-Ting Yuan, Seng-Howe Nguang, Chih-Ming Liang, Shih-Cheng Yang, Cheng-Kun Wu, Pin-I Hsu, Deng-Chyang Wu, Seng-Kee Chuah

**Affiliations:** 1 Division of Gastroenterology, Department of Internal Medicine, Kaohsiung Medical University Hospital and Kaohsiung Medical University, Kaohsiung, Taiwan; 2 Department of Pharmacy, Kaohsiung Chang Gung Memorial Hospital, Kaohsiung, Taiwan; 3 School of Pharmacy, Kaohsiung Medical University, Kaohsiung, Taiwan; 4 Division of Hepato-gastroenterology; Department of Internal Medicine, Kaohsiung Chang Gung Memorial Hospital, Kaohsiung, Taiwan; 5 Chang Gung University, College of Medicine, Kaohsiung, Taiwan; 6 Division of Gastroenterology; FooYin University Hospital, Pin-Tung, Taiwan; 7 Division of Hepato-gastroenterology; Department of Internal Medicine, Buddist Tzu Chi General Hospital, Dalin Branch, Taiwan; 8 Divisions of Gastroenterology, Yuan General Hospital, Kaohsiung, Taiwan; 9 Division of Gastroenterology; Pin-Tung Christian Hospital, Pin-Tung, Taiwan; 10 Division of Gastroenterology, Department of Internal Medicine, Kaohsiung Veterans General Hospital, National Yang-Ming University, Kaohsiung, Taiwan; Istituto Di Ricerche Farmacologiche Mario Negri, ITALY

## Abstract

The association of *Helicobacter pylori* eradication with the occurrence of renal dysfunction in patients with peptic ulcer diseases is still unclear. This study aimed to clarify the relevance of *H*. *pylori* eradication to the occurrence of chronic kidney diseases in patients with peptic ulcer diseases. Data that were available from 2000–2011 were extracted from the National Health Insurance Research Database in Taiwan, and all patients with peptic ulcer diseases (n = 208 196) were screened for eligibility. We divided randomly selected patients into an *H*. *pylori* eradication cohort (cohort A, n = 3593) and matched them by age and sex to a without *H*. *pylori* eradication cohort (cohort B, n = 3593). Subgroup analysis was further performed for *H*. *pylori* eradication within ≤ 90 days of the diagnosis date (early eradication, n = 2837) and within 91–365 days (non-early eradication, n = 756). Cox proportional hazards regression analysis was used to estimate the association of *H*. *pylori* eradication with the risk of developing chronic kidney diseases and mortality. We observed that there were more patients suffering from chronic kidney disease in cohort B than in the early eradication subgroup of cohort A (8.49% vs. 6.70%, respectively, *p = 0*.*0075*); the mortality rate was also higher in cohort B (4.76% vs. 3.70%, respectively, *p = 0*.*0376*). Old age, pulmonary disease, connective tissue disorders, and diabetes were risk factors for chronic kidney diseases but early *H*. *pylori* eradication was a protective factor against chronic kidney diseases (hazard ratio: 0.68, 95% confidence interval: 0.52–0.88, *p = 0*.*0030*), and death (hazard ratio: 0.69, 95% confidence interval: 0.49–0.96, *p = 0*.*0297*). In conclusion, our findings have important implications suggesting that early *H*. *pylori* eradication is mandatory since it is associated with a protective role against the occurrence of chronic kidney diseases.

## Introduction

*Helicobacter pylori* is a spiral-shaped, microaerophilic Gram-negative flagellate bacterium that usually resides in the gastric mucosa [1, 2]. *H*. *pylori* infection is a common bacterial infection of humans worldwide. Approximately 50% of the world’s population is colonized with *H*. *pylori*, and the infection levels exceed 70% in some developing areas [3, 4]. An association between *H*. *pylori* infection and the development of gastrointestinal diseases, such as peptic ulcer, gastric hyperplastic polyps, gastric adenoma, gastric cancer, and gastric mucosa associated-lymphoid tissue lymphoma, has been demonstrated [5, 6].

In addition, several studies have reported that the development of some extragastrointestinal disorders, including idiopathic thrombocytopenic purpura, chronic idiopathic urticaria, iron deficiency anemia, ischemic heart diseases, modified lipid profiles, insulin resistance, and neurodegenerative diseases is closely linked with *H*. *pylori* infection of the gastric mucosa [7–12].

However, the relevance of *H*. *pylori* infection and eradication to renal dysfunction is still unclear. The results of a previous study suggested that *H*. *pylori* infected patients with concomitant chronic kidney disease (CKD) and cardiovascular diseases risk factors were at higher risk of end stage renal disease (ESRD) than those with a singer factor [13]. However, little is known about whether eradication of the bacteria has any effect on renal function. Therefore, this nationwide cohort study aimed to investigate the association of *H*. *pylori* eradication with the occurrence of chronic kidney diseases in patients with peptic ulcer diseases (PUD).

## Materials and Methods

### Ethics Statement

The study protocol was approved by the institutional review board and the Ethics Committee of Chang Gung Memorial Hospital and Kaohsiung Medical University Hospital, Kaohsiung, Taiwan. The Ethics Committee waived the requirement for informed consent for this study, and all of the data were analyzed anonymously.

### Data Source

We used a database of a million patients who were randomly selected for analysis from 22.6 million of Taiwan’s National Health Insurance (NHI) enrollees in 2000–2011 (NHI 2000). The Taiwan NHI was created by the Taiwan government as a single-payer health insurance program on March 1, 1995 [14]. The diagnoses used in the National Health Insurance Research Database (NHIRD) are coded according to the diagnostic criteria of the International Classifications of Diseases, Revision 9, Clinical Modification (ICD-9-CM). The data analysts were staff of Kaohsiung Medical Center, a site of the Collaboration Center of Health Information Application, Ministry of Health and Welfare. The cohort dataset of a million randomly selected individuals and the dataset of patients with recorded illnesses included individuals who were still alive in 2011. The recorded data for each individual included the enrollment files, claims data, serious illness files, and the drug prescription registry. In the cohort dataset, each patient’s original identification number was anonymized and de-identified prior to retrieval of data for privacy purposes.

### Study Subjects

**Fig 1:**
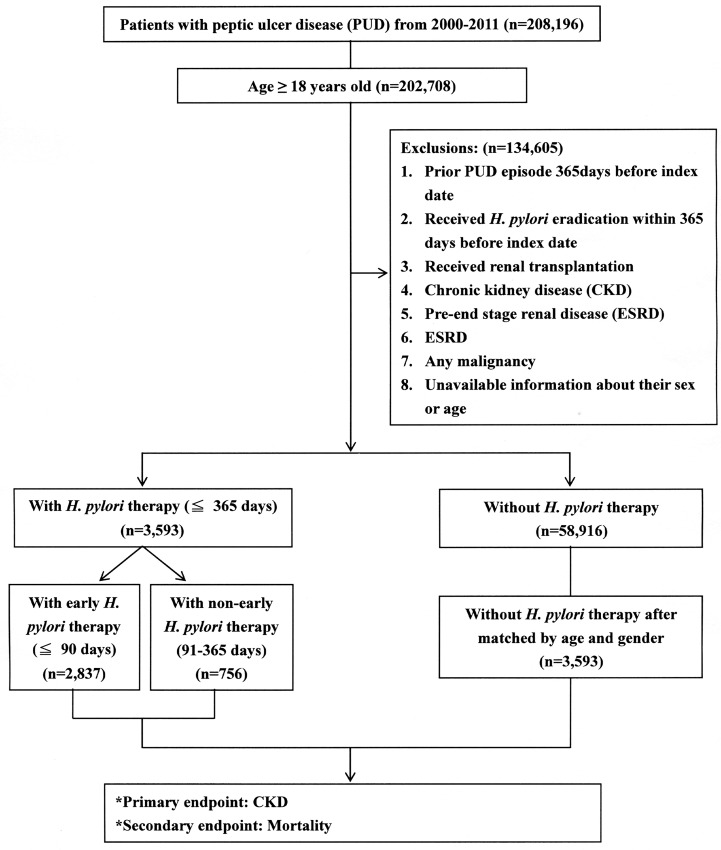
Schematic flowchart of study design.

In this population-based cohort study, patients with PUD (n = 208 196) were screened for eligibility, and those aged more than 18 years old were included (n = 202 708). Fig 1 shows the schematic flowchart of the study design. We used ICD-9-CM codes (531–534) to identify patients with PUD. The date of diagnosis with PUD was used as the index date. Patients who underwent *H*. *pylori* eradication within 365 days before the index date, patients who received renal transplantation (ICD-9-CM code V420), and patients who were diagnosed with prior PUD, CKD, pre-ESRD, ESRD (ICD-9-CM code 585), any malignancy, or had unavailable information about their sex or age were all excluded (n = 134 605).

We used ICD-9-CM codes to identify renal transplantation and CKD patients who were hospitalized at least once or presented for two or more outpatient visits at least 84 days apart. Patients who used erythropoietin (anatomical therapeutic chemical codes) or underwent arteriovenous shunt creation (ICD-9-CM codes 4470, details of inpatient orders codes 69032C and 69034C) were defined as pre-ESRD. Patients who received hemodialysis or peritoneal dialysis for at least 3 months were defined as ESRD.

We divided the patients into those with *H*. *pylori* eradication (cohort A, n = 3593) and without *H*. *pylori* eradication (n = 58916), and selected the same number of patients in cohort A from the non-eradication cohort to form the comparison cohort (cohort B, n = 3593) after matching by age and sex. Patients with *H*. *pylori* eradication performed within ≤ 365 days of the index date were included in cohort A.

*H*. *pylori* eradication triple or quadruple therapy was defined as proton-pump inhibitor (PPI) or histamine type 2 receptor antagonists (H_2_RA) plus clarithromycin or metronidazole plus amoxicillin or tetracycline, with or without bismuth. These drug combinations were prescribed within the same prescription order, and the duration of therapy was 7–14 days. Subgroup analysis was further performed according to the timing of *H*. *pylori* eradication after initial diagnosis. Early *H*. *pylori* eradication was defined as treatment ≤ 90 days after the index date (n = 2837) and non-early eradication was defined as those who received treatment > 90 days but ≤ 365 days after the index date (n = 756).

### Comorbidities and Other Covariates

General health status was assessed by the Charlson co-morbidity index (CCI), which is the sum of the weighted scores of 17 co-morbid conditions and is widely used to control for confounding in epidemiological studies [15]. Exposure to nephrotoxic drugs such as nonsteroidal anti-inflammatory drugs (NSAIDs), angiotensin converting enzyme inhibitors (ACEI), and angiotensin II receptor blockers (ARB) was defined as a patient having a prescription for any of them at least 1 day after the index date through the occurrence of any event related to this study, withdrawal from the NHI, the end of the study period, or death, whichever came first. The NHIRD database contains the details of every prescription, including the doses, frequencies, dates, and administration routes.

### Outcome Measurements

The primary endpoint of this study was newly diagnosed CKD and the secondary endpoint was all-cause mortality. Newly diagnosed CKD was defined as having at least one record of CKD during hospitalization or during two or more outpatient visits that occurred at least 84 days apart.

### Statistics

Categorical variables are presented as percentages. The *X*^*2*^ test was used for categorical data. Cox proportional hazards regression analysis was used to estimate the association of *H*. *pylori* eradication with the risk of CKD and mortality. The Cox proportional hazards model was used to estimate the age-, sex-, comorbidity-, and nephrotoxicity drug-specific hazard ratio (HR) and 95% confidence interval (CI). We also used Kaplan-Meier curves to display the association of *H*. *pylori* eradication to the occurrence of CKD and mortality over time. All statistical analyses were conducted using the statistical software package SAS (version 9.3; SAS Institute Inc., Cary, NC, USA). A two-sided *p* value < 0.05 was considered significant.

## Results

### Demographic Data

The demographic data of the two patient cohorts after matching by age and sex are shown in Table 1. A total of 68103 participants with PUD met the inclusion criteria. Most patients in cohort A received first line *H*. *pylori* eradication therapy (n = 3562, 99.14%). The mean ages of the patients in both cohort A and cohort B were 50.15 ± 15.13 years (*p = 1*.*0000*) and 57.72% of cohort A and cohort B were male (*p = 1*.*0000*). The Charlson scores in cohort A and cohort B patients were 0.52 ± 0.71 and 0.34 ± 0.61, respectively (*p < 0*.*0001*). There were no significant differences in comorbidities or nephrotoxicity drug use between the two cohorts.

**Table 1:** Demographic characteristics of the study population with and without HP therapy after matched by age and gender.

Characteristics	Cohort A		Cohort B		*P* value
	Patients with HP therapy (≦ 365 days) (n = 3593)		Patients without HP therapy (n = 3593)		
	N	%	N	%	
HP therapy*					
**First**	3562	99.14%	—	—	
HP4+HP3+HP1	3491	98.01%	—	—	
HP4+HP3+HP2	3	0.08%	—	—	
HP5+HP3+HP1	116	3.26%	—	—	
HP5+HP3+HP2	1	0.03%	—	—	
**Second**	32	0.89%	—	—	
HP4+HP6+HP8+HP2	0	0.00%	—	—	
HP5+HP6+HP8+HP2	0	0.00%	—	—	
HP4+HP7+HP1	28	87.50%	—	—	
HP5+HP7+HP1	9	28.13%	—	—	
**Age, years (mean±SD)**	50.15±15.13		50.15±15.13		*1*.*0000*
**Age_Class1**					
< 49	1863	51.85%	1848	51.43%	*0*.*9754*
50–59	803	22.35%	802	22.32%	
60–69	480	13.36%	491	13.67%	
≥ 70	447	12.44%	452	12.58%	
**Age_Class2**					
< 65	2926	81.44%	2922	81.32%	*0*.*9035*
≥ 65	667	18.56%	671	18.68%	
**Gender**					
Male	2074	57.72%	2074	57.72%	*1*.*0000*
Female	1519	42.28%	1519	42.28%	
**Charlson score**					
0	2574	71.64%	2085	58.03%	*< .0001*
1	843	23.46%	1223	34.04%	
2	148	4.12%	234	6.51%	
≥ 3	28	0.78%	51	1.42%	
**Charlson score (mean±SD)**	0.52±0.71		0.34±0.61		*< .0001*
**Charlson comorbidity**					
Acute myocardial infarction	0	0.00%	0	0.00%	*—*
Congestive heart failure	5	0.14%	5	0.14%	*1*.*0000*
Peripheral vascular disease	0	0.00%	0	0.00%	*—*
Cerebral vascular accid	95	2.64%	106	2.95%	*0*.*4313*
Dementia	5	0.14%	5	0.14%	*1*.*0000*
Pulmonary disease	124	3.45%	124	3.45%	*1*.*0000*
Connective tissue disorder	11	0.31%	11	0.31%	*1*.*0000*
Peptic ulcer	—	—	—	—	*—*
Liver disease	151	4.20%	151	4.20%	*1*.*0000*
Diabetes	153	4.26%	159	4.43%	*0*.*7284*
Diabetes complications	33	0.92%	28	0.78%	*0*.*5203*
Paraplegia	0	0.00%	0	0.00%	*—*
Renal disease	0	0.00%	0	0.00%	*—*
Cancer	0	0.00%	0	0.00%	*—*
Metastatic cancer	0	0.00%	0	0.00%	*—*
Severe liver disease	0	0.00%	0	0.00%	*—*
HIV	2	0.06%	0	0.00%	*0*.*1572*
**Comorbidity**					
Hypertension	577	16.06%	577	16.06%	*1*.*0000*
Diabetes	201	5.59%	201	5.59%	*1*.*0000*
Hyperlipidemia	224	6.23%	224	6.23%	*1*.*0000*
Coronary artery disease	144	4.01%	144	4.01%	*1*.*0000*
**Acute kidney injury**	4	0.11%	0	0.00%	*0*.*0454*
**Nephrotoxicity drug**					
NSAIDs	599	16.67%	599	16.67%	*1*.*0000*
ACEI/ARB	298	8.29%	298	8.29%	*1*.*0000*

Abbreviations: HP, Helicobacter pylori; HIV, human immunodificiency virus; NSAIDs, nonsteroidal anti-inflammatory drugs; ACEI, angiotensin converting enzyme inhibitor; ARB, angiotensin II receptor blocker

*HP1 = Amoxicillin, HP2 = Metronidazole, HP3 = Clarithromycin, HP4 = PPI, HP5 = H2 blockers, HP6 = Bismuth, HP7 = Levofloxacin, HP8 = Tetracycline

### Outcomes of the Study Population

Table 2 summarizes the occurrences of CKD and the mortality rate in both cohorts. The results show that more patients suffered from the occurrence of CKD in cohort B than those in cohort A who received early *H*. *pylori* eradication (8.49% vs. 6.70%, respectively, *p = 0*.*0075*); the mortality rate was also higher in cohort B (4.76% vs. 3.70%, respectively, *p = 0*.*0376*). However, when we compared cohort B to all those in cohort A who received *H*. *pylori* eradication, there was no significant difference in CKD occurrence (8.49% vs. 7.88%, respectively, *p = 0*.*3437*) or mortality rate (4.76% vs. 4.51%, respectively, *p = 0*.*6135*).

**Table 2:** Outcomes of the study population.

**Characteristics**	**Patients with HP therapy (≦ 365 days) (n = 3593)**		**Patients without HP therapy (n = 3593)**		***P* value**
	**N**	**%**	**N**	**%**	
**Endpoint**					
Primary-CKD	283	7.88%	305	8.49%	*0*.*3437*
Death	162	4.51%	171	4.76%	*0*.*6135*
**Characteristics**	**Patients with early HP therapy (≦ 90 days) (n = 2837)**		**Patients without HP therapy (n = 3593)**		***P* value**
	**N**	**%**	**N**	**%**	
**Endpoint**					
Primary-CKD	190	6.70%	305	8.49%	*0*.*0075*
Death	105	3.70%	171	4.76%	*0*.*0376*

Abbreviations: HP, *Helicobacter pylori*; CKD: chronic kidney disease

### Multivariate Analysis

By Cox proportional hazard regression analysis, *H*. *pylori* eradication was not a significant protective factor against CKD (HR: 1.02, 95% CI: 0.86–1.20, *p = 0*.*8349*) or death (HR: 1.05, 95% CI: 0.84–1.32, *p = 0*.*6511*) after adjusting for age, sex, Charlson score, and nephrotoxicity drug use (Tables 3 and 4). However, older age, pulmonary disease, connective tissue disorders, and diabetes were risk factors for CKD. In the mortality analysis, older age, male sex, congestive heart failure, connective tissue disorders, liver disease, diabetes, and acute kidney injury were risk factors for death.

**Table 3:** Multivariate analysis of potential risk factors for the occurrence of CKD in patients with PUD (with and without HP therapy).

Variable	Multivariate analysis			
	HR	95% CI		*P* value
**Group**				
Patients without HP therapy	1			
Patients with HP therapy (≦ 365 days)	1.02	0.86	1.20	*0*.*8349*
**Age**	1.05	1.04	1.06	*< .0001*
**Gender (male is reference)**	1.10	0.93	1.30	*0*.*2511*
**Charlson comorbidity**				
Congestive heart failure	1.61	0.50	5.15	*0*.*4223*
Cerebral vascular accid	0.90	0.63	1.29	*0*.*5613*
Dementia	1.08	0.34	3.44	*0*.*8949*
Pulmonary disease	0.65	0.43	0.99	*0*.*0460*
Connective tissue disorder	4.18	1.71	10.22	*0*.*0017*
Peptic ulcer	1.01	0.84	1.22	*0*.*8916*
Liver disease	1.14	0.77	1.69	*0*.*5068*
**Comorbidity**				
Hypertension	1.19	0.93	1.52	*0*.*1639*
Diabetes	2.25	1.76	2.87	*< .0001*
Hyperlipidemia	0.80	0.57	1.10	*0*.*1689*
Coronary artery disease	1.02	0.74	1.40	*0*.*9192*
**Acute kidney injury**	1.51	0.21	10.87	*0*.*6846*
**Nephrotoxicity drug**				
NSAIDs	0.94	0.75	1.16	*0*.*5540*
ACEI/ARB	1.21	0.90	1.63	*0*.*2081*

Abbreviations: CKD: chronic kidney disease; PUD: peptic ulcer disease; HP: *Helicobacter pylor*i; HR: hazard ratio; CI: confidence interval; NSAIDs: nonsteroidal anti-inflammatory drugs; ACEI: angiotensin converting enzyme inhibitor; ARB: angiotensin II receptor blocker

**Table 4:** Multivariate analysis of potential risk factors for mortality in patients with PUD (with and without HP therapy).

Variable	Multivariate analysis			
	HR	95% CI		*P* value
**Group**				
Patients without HP therapy	1			
Patients with HP therapy (≦ 365 days)	1.05	0.84	1.32	*0*.*6511*
**Age**	1.07	1.07	1.08	*< .0001*
**Gender (male is reference)**	0.75	0.60	0.94	*0*.*0110*
**Charlson comorbidity**				
Congestive heart failure	1.60	1.10	2.33	*0*.*0150*
Cerebral vascular accident	1.89	0.58	6.14	*0*.*2872*
Dementia	0.99	0.62	1.59	*0*.*9776*
Pulmonary disease	1.65	0.23	11.96	*0*.*6205*
Connective tissue disorder	0.63	0.49	0.82	*0*.*0006*
Peptic ulcer	1.21	0.70	2.09	*0*.*4913*
Liver disease	1.60	1.10	2.33	*0*.*0150*
**Comorbidity**				
Hypertension	1.19	0.87	1.63	*0*.*2806*
Diabetes	2.04	1.48	2.81	*< .0001*
Hyperlipidemia	0.86	0.55	1.34	*0*.*5005*
Coronary artery disease	1.02	0.68	1.53	*0*.*9438*
**Acute kidney injury**	4.93	1.19	20.41	*0*.*0277*
**Nephrotoxicity drug**				
NSAIDs	0.75	0.56	1.00	*0*.*0507*
ACEI/ARB	1.13	0.78	1.65	*0*.*5184*

Abbreviations: CKD: chronic kidney disease; HP: *Helicobacter pylor*i; HR: hazard ratio; CI: confidence interval; NSAIDs: nonsteroidal anti-inflammatory drugs; ACEI: angiotensin converting enzyme inhibitor; ARB: angiotensin II receptor blocker

When we performed subgroup analysis to look at the possible effect of the timing of *H*. *pylori* eradication, we found that early *H*. *pylori* eradication was a protective factor against CKD (HR: 0.68, 95% CI: 0.52–0.88, *p = 0*.*0030*), and death (HR: 0.69, 95% CI: 0.49–0.96, *p = 0*.*0297*) compared to non-early *H*. *pylori* eradication (Tables 5 and 6).

**Table 5:** Multivariate analysis of potential risk factors for the occurrence of CKD in patients with PUD (with early and non-early HP therapy).

Variable	Multivariate analysis			
	HR	95% CI		*P* value
**Group**				
Patients with HP therapy (91–365 days)	1			
Patients with HP therapy (≦ 90 days)	0.68	0.52	0.88	*0*.*0030*
**Age**	1.05	1.04	1.05	*< .0001*
**Gender (male is reference)**	1.10	0.86	1.39	*0*.*4525*
**Charlson comorbidity**				
Congestive heart failure	1.53	0.21	11.31	*0*.*6754*
Cerebral vascular acid	0.94	0.55	1.60	*0*.*8093*
Dementia	3.79	1.08	13.23	*0*.*0371*
Pulmonary disease	0.82	0.48	1.42	*0*.*4839*
Connective tissue disorder	4.35	1.36	13.94	*0*.*0134*
Peptic ulcer	0.91	0.67	1.23	*0*.*5315*
Liver disease	1.30	0.76	2.25	*0*.*3425*
**Comorbidity**				
Hypertension	1.18	0.83	1.69	*0*.*3554*
Diabetes	2.07	1.44	2.97	*< .0001*
Hyperlipidemia	0.67	0.40	1.12	*0*.*1239*
Coronary artery disease	1.24	0.79	1.96	*0*.*3438*
**Acute kidney injury**				
**Nephrotoxicity drug**	1.08	0.79	1.47	*0*.*6327*
NSAIDs	1.13	0.73	1.76	*0*.*5827*
ACEI/ARB	1.53	0.21	11.31	*0*.*6754*

Abbreviations: CKD: chronic kidney disease; PUD: peptic ulcer disease; HP: *Helicobacter pylor*i; HR: hazard ratio; CI: confidence interval; NSAIDs: nonsteroidal anti-inflammatory drugs; ACEI: angiotensin converting enzyme inhibitor; ARB: angiotensin II receptor blocker

**Table 6:** Multivariate analysis of potential risk factors for mortality in patients with PUD (with early and non-early HP therapy).

Variable	Multivariate analysis			
	HR	95% CI		*P* value
**Group**				
Patients with HP therapy (91–365 days)	1			
Patients with HP therapy (≦ 90 days)	0.69	0.49	0.96	*0*.*0297*
**Age**	1.08	1.07	1.09	*< .0001*
**Gender (male is reference)**	0.71	0.52	0.99	*0*.*0413*
**Charlson comorbidity**				
Congestive heart failure	1.56	0.89	2.72	*0*.*1204*
Cerebral vascular accident	1.85	0.41	8.41	*0*.*4283*
Dementia	1.00	0.52	1.92	*0*.*9950*
Pulmonary disease	3.72	0.50	27.47	*0*.*1984*
Connective tissue disorder	0.78	0.51	1.17	*0*.*2251*
Peptic ulcer	1.21	0.56	2.62	*0*.*6345*
Liver disease	1.56	0.89	2.72	*0*.*1204*
**Comorbidity**				
Hypertension	1.11	0.71	1.73	*0*.*6558*
Diabetes	2.68	1.74	4.14	*< .0001*
Hyperlipidemia	1.03	0.56	1.87	*0*.*9322*
Coronary artery disease	0.98	0.54	1.79	*0*.*9524*
**Acute kidney injury**				
**Nephrotoxicity drug**	0.89	0.60	1.32	*0*.*5450*
NSAIDs	1.07	0.63	1.83	*0*.*8017*
ACEI/ARB	0.89	0.60	1.32	*0*.*5450*

Abbreviations: CKD: chronic kidney disease; HP: *Helicobacter pylor*i; HR: hazard ratio; CI: confidence interval; NSAIDs: nonsteroidal anti-inflammatory drugs; ACEI: angiotensin converting enzyme inhibitor; ARB: angiotensin II receptor blocker

### Kaplan-Meier Analysis

**Fig 2:**
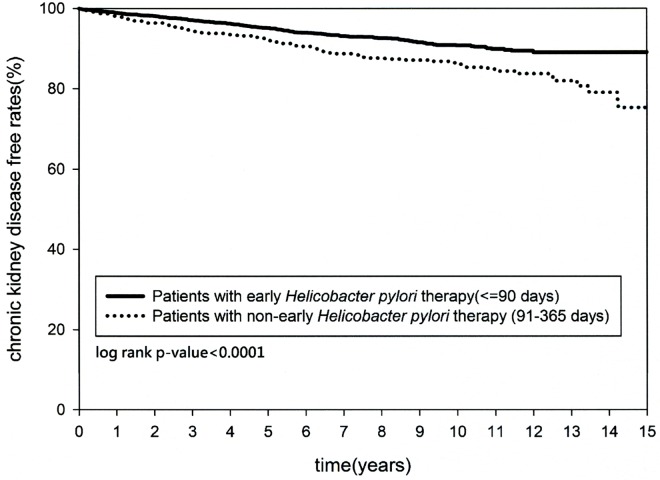
Kaplan-Meier curve for cumulative chronic kidney disease rate between patients with early and non-early *Helicobacter pylori* therapy.

**Fig 3:**
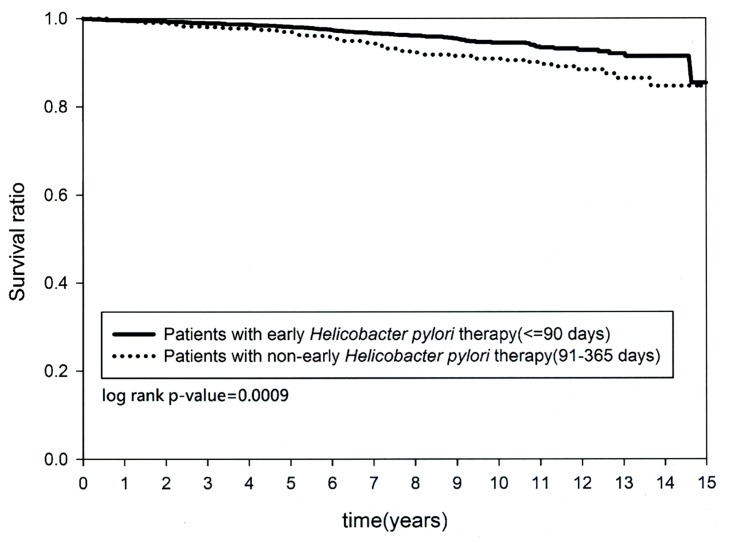
Kaplan-Meier curve for cumulative mortality rate between patients with early and non-early *Helicobacter pylori* therapy.

Both the cumulative occurrence of CKD and the mortality rate were not significantly different (*p = 0*.*8834* and *p = 0*.*5132*, respectively) between cohort A and cohort B at the last follow-up since the index date. On the other hand, the cumulative occurrence of CKD and the mortality rate were significantly different in the patients with early *H*. *pylori* eradication compared with non-early *H*. *pylori* eradication (*p < 0*.*0001* and *p = 0*.*0009* respectively) (Figs 2 and 3).

## Discussion

There are reports on the association between *H*. *pylori* infection and ESRD but evidence of an effect of *H*. pylori eradication on kidney function is seldom reported. This study aimed to clarify the relevance of *H*. *pylori* eradication to the occurrence of chronic kidney diseases in patients with peptic ulcer diseases. Our study observed that *H*. *pylori* eradication within 90 days of diagnosis was associated with decreased rates of occurrence of CKD and mortality compared with those without early *H*. *pylori* eradication.

Several studies have proven that gastric and extra-gastric *H*. *pylori* infection plays a role in the development of systemic disease such as renal dysfunction [16]. In addition, a retrospective cohort study reported that *H*. *pylori* infection may be a risk factor for subsequent ESRD but the authors did not investigate the possibility that eradication of the bacteria could be a protective factor [13]. Nozaki et al. found that *H*. *pylori* eradication at an early stage of inflammation (< 15 weeks) might be effective in preventing gastric carcinogenesis [17]. This might also imply that the timing of eradication could be crucial in minimizing the damage from inflammatory events initiated by *H*. *pylori*.

We defined the early *H*. *pylori* eradication therapy cohort as patients who received therapy within 90 days of initial diagnosis. The observations about preventing inflammation could partly explain the observation in the current study that early eradication of *H*. *pylori* was associated with a lower rate of occurrence of CKD as compared to those infected PUD subjects who did not received *H*. *pylori* eradication or had non-early eradication, after adjusting for age, sex, co-morbidities, and nephrotoxicity drugs.

The presence of *H*. *pylori* is strongly associated with PUD. It has been found that *H*. *pylori* exists in > 90% of duodenal ulcer patients and 70–90% of gastric ulcer patients [18]. Before adjusting for confounding factors, we found that there was no significant difference in CKD or mortality rates between patients with *H*. *pylori* eradication ≤ 365 days of the index date and those without *H*. *pylori* eradication, whereas a significant decrease in the occurrence of CKD and mortality was noted for patients in the early *H*. *pylori* eradication group compared to those without *H*. *pylori* eradication. These results were similar after we adjusted for confounding factors. Previously, Wu et al. proved early *H*. *pylori* eradication was an independent protective factor against gastric cancer [19]. Likewise, we observed that early *H*. *pylori* eradication played a role in renoprotection in the current study.

It has also been reported that *H*. *pylori* infection can contribute to endothelial dysfunction, which is related to CKD development and renal function decline [20–22]. A possible mechanism for this could be that chronic *H*. *pylori* infection might induce a persistent systemic and vascular inflammation and hence result in the malabsorption of folate, vitamin B6, and vitamin B12, leading to failure of methylation by 5-methyl-tetrahydrofolic acid and, thus, to hyperhomocysteinanemia, which causes toxicity to endothelial cells. Moreover, it is also possible that *H*. *pylori* infection increases asymmetric dimethylarginine (ADMA) levels, causing deep metabolic modifications [23]. High plasma ADMA levels have been shown to contribute to the development of oxidative stress and interstitial and glomerular fibrosis, which are associated with endothelial dysfunction and CKD progression [24].

The optimal timing for eradication is an important issue. Decreasing *H*. *pylori* exposure duration would shorten the period of the above pathophysiologic processes, so we can infer that early *H*. *pylori* eradication would be associated with lower risks of CKD development.

Interestingly, our study also observed that pulmonary disease and connective tissue disorders could be related to CKD development. Similar results were reported for two other cohort studies. Chen et al. found that chronic obstructive pulmonary disease was a risk factor for the development of CKD [25]. Chiu et al. observed rheumatoid arthritis patients had a higher risk of developing CKD [26]. These findings imply that comorbidities could be additive factors for the occurrence of CKD.

The strength of our study is its large sample size obtained by enrollment of a nationally representative cohort. Detailed information regarding *H*. *pylori* eradication therapy, NSAIDs, ACEI, and ARB were obtained by linking to the NHI pharmacy database under the Reimbursement Policy requested by NHI to reduce the possibility of duplication or misclassification. Furthermore, many important covariates such as the underlying diseases were available in detail.

On the other hand, it is inevitable that our study has several limitations. First, *H*. *pylori* eradication has had a relatively high failure rate over the years in Taiwan [27–29]. However, the Taiwanese patients enrolled during our study period who received first line *H*. *pylori* therapy could be expected to achieve a > 90% eradication rate with standard triple therapy of prescriptions of twice daily treatment with PPI combined with clarithromycin 500 mg and amoxicillin 1 g for 1 week [30]. This high rate is probably due to the low clarithromycin drug resistance rate at the time. Second, we were unable to assess several important risk factors of CKD related to lifestyle such as obesity or cigarette smoking because this information was not recorded in the NHIRD database. Finally, we studied a population largely consisting of people from Han Chinese descent, so our results might not be generalizable to non-Asians.

In conclusion, our findings have important implications, suggesting that early *H*. *pylori* eradication ≤ 90 days of the index date is mandatory since it is associated with a protective role against the occurrence of chronic kidney diseases. Further studies, especially population-based studies, will be helpful to confirm our results.

## Supporting Information

S1 Dataset This file provides all data of the manuscript. (XLS)

## Abbreviations

*H. pylori*: *Helicobacter pylori*
CKD: chronic kidney disease
ESRD: end stage renal disease
PUD: peptic ulcer diseases
NHI: National Health Insurance
NHIRD: National Health Research Institute database
ICD-9-CM: International Classifications of Diseases, Revision 9, Clinical Modification
PPI: proton pump inhibitors
H_2_RA: histamin-2 receptor antagonists
CCI: Charlson co-morbidity index
NSAIDs: nonsteroidal anti-inflammatory drugs
ACEI: angiotensin converting enzyme inhibitor
ARB: angiotensin II receptor blocker
HR: hazard ratio
CI: confidence interval
ADMA: asymmetric dimethylarginine
SD: standard deviation
HIV: human immunodificiency virus
